# Antibiotics for *Gram*-negative infections

**DOI:** 10.1590/S1679-45082015ED3451

**Published:** 2015

**Authors:** Jacyr Pasternak

**Affiliations:** 1 Hospital Israelita Albert Einstein, São Paulo, SP, Brazil.

We do need them, and we need them now! New antibacterial drugs have been developed on the last 2 years for *Gram*-positive agents,^1^ but not with new targets. There has been real progress in developing antimycobacterial agents, including some new modes of action;^2^ however, what we have available today for *Gram*-negative multiresistant bacteria is the same we had 5 years ago, except for some molecular alterations in antibiotics, but no new bacterial target. Ceftazidime plus avibactam is combination therapy using a new beta-lactamase inhibitor, and similar combinations are also available in developed countries, as ceftozalone plus tazobactam,^4^ and meropenem plus RPX7009.^3^ The new aminoglycoside being tested is plazomycin,^4^ and there is a new quinolone, delafloxacin.^5^ Very old agents are being resurrected, such as fosfomycin,^6^ and only recently has been discovered what could be called a completely new type of antibacterial agent, teixobactin.^7^

We do know the whole genome of many *Gram*-negative rods, but knowledge of the genetic make up of those germs did not lead to new imaginative antimicrobial agents. The golden era of antibiotic discovery was between the first discovery, that is, penicillin, in 1940, and the 1980’s; since them there was a dearth of new antibiotic discoveries. There are some reasons for this: first, the low hanging fruits have been collected, the easy discoveries have been made and the easy to find fungii have been studied to verify their capabilities to make antibiotics. Second, the financial incentives to the large pharmaceutical companies regarding antibiotics have never been very favourable. One cannot compare the revenues from antibiotics to gross revenues from chronic use drugs, such as statins or sildenafil and its derivatives. The same rationale applies to antineoplastic agents, which are also used for relatively long time and can be very expensive. Moreover, the new immunobiologics are financially worth the effort of being developed, both to auto-immune and neoplastic diseases. When wisely prescribed, antibiotics will be used for at most 2 to 3 weeks. On the other hand, statins are used for decades; and sildenafil and its derivatives, if they work (and mostly do), will be used by males for as much as they breathe…Third, there are more regulatory restrictions to develop antibiotics. There are relevant reasons for that: after being used in clinical practice, some quinolones proved to be hazardous and had to be recalled,^8^ with real damages to financial and to the image of pharmaceutical companies.

Those motives do not justify the long time to evaluate and appreciate the phase 1, 2 and 3 for antibiotics being developed. The Americans complain of the *Food and Drug Administration* (FDA) for those long delays between clinical studies and authorization to use the drugs, but they do not experience our National Health Surveillance Agency (ANVISA* - Agência Nacional de Vigilância Sanitária),* and have no idea how lucky they are…

Just one example, in order to authorize the use of a drug in Brazil, ANVISA requires its representatives to inspect the manufacturing plant, even if the sites have been already inspected and approved by the FDA. What our brave Brazilian officers look at and inspect (and most do not speak another language apart from Portuguese) is a mystery…

To make things even more complicated, no new antibiotics can be tested in Brazil for research purposes, if not evaluated by the National Research Ethics Committee* (*CONEP *- Comissão Nacional de Ética em Pesquisa).* This national review board is typically Brazilian, like *jabuticabas* and turtles found on tree branches… The committee staff is convinced that all studies carried out by multinational pharmaceutical industries, in less developed countries, are suspicious. In addition, they take long to appreciate the vast documentation required, they are picky regarding informed consent forms and other details of research proposals, making paperwork go forth and back for months. If somebody proposes some research with flimsy justification using Brazilian flora products they are much more permissive… With both institutions working in tandem in Brazil, we are always late to obtain new drugs, and lag behind in developing expertise to use them.

What all these mean? Patients could benefit from new antibiotics and they do not; Brazilian physicians interested in clinical research give up after experiencing all difficulties; and a universal problem − lack of new anti *Gram*-negative agents − will be even worse around here. Furthermore, our *Gram*-negative rods are graduating in all types of resistance. KPC is very common at our hospitals and our *Pseudomonas aeruginosa* has some strains that do not respond to any known agent.

This situation demands changes, and fast changes. We do not know how, but they must happen…

## References

[1] Morata L, Mensa J, Soriano A (2015). New antibiotics against gram-positives: present and future indications. Curr Opin Pharmacol.

[2] Zumla A, Chakaya J, Centis R, D’Ambrosio L, Mwaba P, Bates M (2015). Tuberculosis treatment and management--an update on treatment regimens, trials, new drugs and adjunct therapies. Lancet Respir Med.

[3] Bettiol E, Harbarth S (2015). Development of new antibiotics: taking off finally?. Swiss Med Wkly.

[4] García-Salguero C, Rodríguez-Avial I, Picazo JJ, Culebras E (2015). Could plazomicin alone or in combination be a therapeutical option against carbapenem-resistant Acinetobacter baumanii?. Antimicrobi Agents Chemother.

[5] Siala W, Mingeot-Leclercq MP, Tulkens PM, Hallin M, Denis O, Van Bambeke F (2014). Comparison of the antibiotic activities of Daptomycin, Vancomycin, and the investigational Fluoroquinolone Delafloxacin against biofilms from Staphylococcus aureus clinical isolates. Antimicrob Agents Chemother.

[6] Popovic M, Steinort D, Pillai S, Joukhadar C (2010). Fosfomycin: and old, new friend?. Eur J Clin Microbiol Infect Dis.

[7] Ling LL, Schneider T, Peoples AJ, Spoering AL, Engels I, Colon BP (2015). A new antibiotic kills pathogens without detectable resistance. Nature.

[8] Stahlmann R, Lode H (1999). Toxicity of quinolones. Drugs.

