# Supplementary material for: Antibiotics for Gram-negative infections
Source: Einstein (Sao Paulo). 2015 Jul-Sep;13(3):vii–viii. doi: 10.1590/S1679-45082015ED3451 (PMC4943804; doi:10.1590/S1679-45082015ED3451)
Supplement: Translation

# Translation: Antibióticos para infecções por *Gram*-negativos

**Authors:** Jacyr Pasternak

**Affiliations:** 1 Hospital Israelita Albert Einstein, São Paulo, SP, Brasil.

Precisamos deles, e agora! Nos últimos 2 anos, foram desenvolvidos novos medicamentos antibacterianos contra agentes *Gram*-positivos,^1^ porém sem novos alvos. Houve progresso real em relação aos agentes antimicobacterianos, inclusive alguns com novos mecanismos de ação;^2^ entretanto, para as bactérias *Gram*-negativas multirresistentes, o que temos hoje é o mesmo que estava disponível há 5 anos, com exceção de algumas alterações moleculares nos antibióticos, mas também sem nenhum alvo bacteriano novo. Ceftazidima com avibactam é um tratamento combinado que utiliza um novo inibidor de betalactamase, e combinações semelhantes são comercializadas nos países desenvolvidos, como ceftolozane com tazobactam,^4^ e meropenem com RPX7009.^3^ O novo aminoglicosídeo que está sendo testado é a plazomicina,^4^ e há uma nova quinolona - delafloxacina.^5^ Agentes muito antigos estão sendo ressuscitados, como a fosfomicina,^6^ e, há pouco tempo, foi descoberto o que poderia ser chamado de um tipo completamente novo de agente bacteriano: a teixobactina.^7^

Conhecemos o genoma completo de muitos bastonetes *Gram*-negativos, mas o conhecimento da constituição genética desses microrganismos não resultou em novos agentes antimicrobianos inovadores. A era de ouro da descoberta de antibióticos foi entre a primeira descoberta, a penicilina, em 1940, e a década de 1980; desde então, houve uma escassez de novas descobertas de antibióticos. Há algumas razões para isso: primeiro, os frutos mais acessíveis já foram colhidos, fizeram-se as descobertas mais fáceis e foram estudados os fungos mais fáceis de serem encontrados para verificar sua capacidade de produzir antibióticos. Segundo, os incentivos financeiros para as grandes empresas farmacêuticas na produção de antibióticos nunca foram muito favoráveis. Não dá para se comparar a receita proveniente da produção de antibióticos com a receita financeira bruta proveniente da produção de medicamentos de uso crônico, como as estatinas ou a sildenafila e seus derivados. O mesmo raciocínio se aplica aos agentes antineoplásicos, que também são usados por um período relativamente longo e podem ser extremamente caros. Além disso, vale a pena – financeiramente – investir no desenvolvimento de novos medicamentos imunobiológicos, tanto para doenças autoimunes quanto neoplásicas. Quando prescritos com sensatez, os antibióticos são utilizados por, no máximo, 2 a 3 semanas. Já as estatinas são usadas por décadas, e a sildenafila e seus derivados, se funcionarem (e a maioria o faz), são utilizados por homens enquanto respirarem... A terceira razão é que há mais restrições regulamentares para o desenvolvimento de antibióticos. Há motivos relevantes para isso: algumas quinolonas, após serem introduzidas na prática clínica, apresentaram riscos e tiveram que ser retiradas do mercado,^8^ com danos reais para as finanças e a imagem das indústrias farmacêuticas.

Esses motivos não justificam o longo período de tempo exigido para avaliar e apreciar as fases 1, 2 e 3 dos antibióticos que estão sendo desenvolvidos. Os americanos reclamam da *Food and Drug Administration* (FDA) pela longa demora que há entre os estudos clínicos e a autorização para a utilização dos medicamentos, mas não conhecem a nossa Agência Nacional de Vigilância Sanitária (ANVISA) e não imaginam o quanto são afortunados…

Apenas como exemplo, para autorizar a utilização de um medicamento no Brasil, a ANVISA exige que seus representantes inspecionem o local de produção, mesmo que essas fábricas já tenham sido inspecionadas e aprovadas pela FDA. O que os nossos nobres funcionários brasileiros examinam e inspecionam (a maioria não fala nenhum outro idioma além do português) é um mistério…

Para complicar ainda mais as coisas, nenhum antibiótico novo pode ser testado no Brasil para fins de pesquisa sem a avaliação da Comissão Nacional de Ética em Pesquisa (CONEP). Essa comissão nacional de ética é algo tipicamente brasileiro, como as jabuticabas e as tartarugas que sobem em árvores… Eles estão convictos de que todos os estudos realizados por empresas farmacêuticas multinacionais, em países menos desenvolvidos, são suspeitos. Além disso, são muito lentos na avaliação da infinidade de documentos que exigem, e extremamente minuciosos em relação aos termos de consentimento livre e esclarecido, e a outros detalhes da pesquisa proposta, fazendo com que os papéis sejam encaminhados de um lugar para o outro por meses. Caso seja proposta uma pesquisa com fracas justificativas usando derivados da flora brasileira, eles são bem mais permissivos… Com as duas instituições trabalhando em conjunto aqui no Brasil estamos sempre atrasados na obtenção de novos medicamentos e ainda mais atrasados no desenvolvimento de experiência na utilização deles.

O que isso significa? Que os pacientes poderiam se beneficiar dos novos antibióticos, mas não o fazem; que os médicos brasileiros interessados em pesquisa clínica desistem após todas essas dificuldades; e que um problema universal − a carência de novos antibióticos contra agentes *Gram*-negativos, será pior ainda por aqui. Ademais, nossos bacilos *Gram*-negativos estão se especializando em todos os tipos de resistência. A KPC é muito comum em nossos hospitais, e a nossa *Pseudomonasaeruginosa* tem algumas cepas que não respondem a nenhum agente conhecido.

Isso tem que mudar, e tem que ser rápido. Não sabemos como, mas precisa acontecer…
